# Affordances and Opportunities for Relational Wellness

**DOI:** 10.1111/jmft.70004

**Published:** 2025-02-04

**Authors:** Karl Tomm, Lance Taylor

**Affiliations:** ^1^ Department of Psychiatry University of Calgary Calgary Alberta Canada; ^2^ Calgary Family Therapy Centre Calgary Alberta Canada

**Keywords:** affordances, opportunities, relational patterns, solution focused, therapy initiatives

## Abstract

This paper explores the concept of “affordances” for relational healing that clients implicitly bring with them to therapy. It highlights the therapist's perception of such affordances as a first step toward conceiving of opportunities to take certain initiatives to enable relational wellness. As therapists become increasingly aware of the multiple possibilities that clients afford them to intervene, they are more liable to utilize such opportunities when doing therapy. By sharpening their observational skills to perceive these affordances, therapists may more readily conceive of associated therapeutic opportunities and initiate relevant interventions. We start with some background theory and then present a few vignettes of clinical work to illustrate the application of these concepts in the course of family therapy.

## 1 Introduction

This article is intended to expand a therapist's awareness of options for enabling clients' possibilities for enacting relational wellness. The overall goal is to enable client movement toward healthier relationships. The focus will not be on individual wellness per se but on the well‐being of relationships. However, we assume that the participants in relationships will experience more personal wellness when their relationships are healthier. By orienting our reflections toward one aspect of therapy, namely, co‐constructing greater wellness in interpersonal relationships, our aspirations in writing this paper are limited. We will not endeavor to cover a comprehensive range of therapeutic possibilities for clients who seek help with their troubles. We assume that most therapists, and virtually all systemic family therapists, already have significant skills in enabling relational wellness. They already respond to clients in various ways to support healthier relationships. Our intention is to draw upon such pre‐existing knowledge and hopefully extend it with a few ideas that any therapist could usefully consider when providing therapy.

We begin with a cursory review of Gibson's Theory of Affordances (1977, 1979) and explore its relevance in setting the stage for distinguishing opportunities to take initiatives to intervene therapeutically. We will describe Tomm's Patterns of Interpersonal Interaction (2014) as a potential framework for drawing upon preferred affordances to co‐construct relational wellness. Then we will describe an extension of Simon and Taylor's focus on Opportunities (2014, 2024) to clarify steps for progressing from affordances to therapeutic responses. Finally, we will share a few clinical vignettes demonstrating therapeutic initiatives to open space for more wellness in ongoing relationships, including clients' relationships with themselves.

## 2 Affordances

### 2.1 Gibson's Theory of Affordances

Gibson (1977, 1979) was a psychologist who adopted an ecological stance in describing how a living system interacts within its environment and niche. The *niche* affords a living system certain possibilities for interaction, which may or may not be taken up. Gibson coined the term “affordance” to describe such possibilities and defined a *niche* as a unique set of affordances. An affordance constitutes a specific interactive possibility in a particular context. For instance, a certain layout of the land may afford shelter, or it may afford concealment to the animal. Whether the animal actually uses the affordance (for shelter or concealment) is another matter that will be explored later. What is relevant here is that the affordance pre‐exists the presence of the animal. The affordance is already there. Without an environment that affords certain basic supports for living, the animal would not survive. However, the affordances need to be perceived before they exist for the animal and can be utilized. Hence, the foundational role of perception for living systems to survive and thrive.

Over the last 50 years, the notion of affordances has been applied in a wide variety of fields: ecology, architecture, design, landscape planning, computer–human interfaces, robots, artificial intelligence, and so forth, often with differing interpretations of Gibson's original concept. There is consensus that an affordance is not a characteristic of the animal, such as a “strength” or some capability. What there is debate about is whether an affordance is a feature of the environment, a relationship between the feature and the animal, or both (Rietveld and Kiverstein 2014). We subscribe to the latter perspective, namely both, that affordances are already there *and* can be brought forth by interacting with what is there. This aligns with what has sometimes been referred to as an enactivist stance among philosophers of mind (Thompson 2010; Maturana and Varela 1987).

Some affordances, once perceived, may be evaluated as “positive” in that if they are taken up and used, they enable the survival and well‐being of the animal. Others are “negative” in that they entail danger or risks to survival. For instance, a cliff affords the risk of falling off. Some features of the environment offer both positive and negative affordances. The same cliff could afford the possibility of a pleasant walk along the edge with a fine view as well as the danger of falling. In the process of evolution, living systems have come to conserve the “instinctual knowledge” required to perceive and utilize positive affordances, while avoiding negative ones. In other words, by virtue of their phylogenetic drift within a specific niche, living systems come to “know” how to survive and thrive by perceiving relevant affordances.

Gibson claims that a given set of affordances in the natural environment offers an animal a certain way of life. He cites a range of examples of basic affordances (Gibson 1979, 128–129). Solid surfaces afford posture, locomotion, collision, and manipulation. Detached objects afford their use as tools, utensils, and weapons. The foregoing are examples of animate beings interacting with inanimate objects. The situation becomes much more complex when the interaction within the niche includes interactions with other living systems. Animals afford each other a huge variety of possibilities (e.g., predation, competition, mating, nurturing, and communication) depending on the specifics of the situation. However, the basic requirement of perceiving affordances to survive and thrive remains the same.

Human beings as animals also perceive affordances in their day‐to‐day living; most of the time, responding to these affordances nonconsciously and seamlessly, while acting according to their basic needs and interests. The richest and most elaborate affordances for humans are provided by other humans. One kind of human behavior typically affords another kind of behavior and vice versa. Two or more persons who are talking can offer each other reciprocal affordances very rapidly and at extremely high levels of sophistication. Indeed, the multiplicity of affordances in human interaction is staggering. While Gibson did not focus on social affordances per se, we hold the foundational position that a huge variety of relational affordances are embedded in the sociocultural background of human understandings about relationships and that perception is key to capitalizing on the opportunities they may present. Furthermore, we humans have extended our evolutionary drift to become consciously aware of at least some of the affordances in our niche. We recursively perceive certain affordances in our environment. And becoming aware of selected affordances enhances the possibility of deliberately employing them as opportunities to respond, in our case, therapeutically. Thus, one major focus of this paper will be to encourage the perception of relational affordances that are “already there” in the context of therapy. Once an affordance is perceived, it can be evaluated as positive or negative, and an opportunity can be formulated to interact with it, either by taking up the possibility (when it is positive) or by avoiding it (when it is negative). We will elaborate on opportunities later, but suffice it to say here that if the affordance is not perceived (consciously or nonconsciously), the opportunity will not arise.

There are many affordances in the environment that are not perceived and, therefore, cannot be taken advantage of by the organism. This is especially true among human beings, where there are almost always possibilities for potential interaction that are not recognized. And, in the doing of therapy, the failure to perceive certain affordances can significantly impoverish one's therapeutic contributions. For this reason, supervisors in therapy training programs orient themselves to help their students learn to perceive selective client affordances for therapeutic change, whether they have been so named or not. When students come to see through the supervisor's eyes, as well as their own eyes, they can recognize more therapeutic possibilities (as will be illustrated in vignette 4 below).

### 2.2 Examples of Affordances Pertinent to Therapy

In the therapeutic encounter, the client(s) is part of the therapist's ecology, and the therapist is part of the client's ecology. Both clients and therapists bring with them a vast amount of prior knowledge about human life and relationships. They already have highly developed and complex networks of ideas, beliefs, meanings, and behavioral tendencies before they even meet. Their knowledge, including each person's understanding of social justice, affords certain possibilities within the therapeutic interaction. Among the clients' understandings are some general affordances derived from cultural expectations about the nature of therapy. For instance, clients typically come prepared to afford the therapist opportunities to inquire about very personal and intimate aspects of their lives and experiences. In turn, therapists typically afford the clients empathic understanding and emotional safety by offering confidentiality and unconditional positive regard (Rogers 1956). The reciprocal coupling of these general affordances in the therapeutic encounter probably contributes to the conditions for the effectiveness of the so‐called “common factors” in therapy (Duncan and Miller 2000; Sprenkle, Davis, and Lebow 2009).

Beyond these common factors, different schools of therapy highlight specific kinds of affordances in their respective approaches. For instance, in the domain of narrative therapy, special attention is given to the idea that clients implicitly afford the therapist opportunities to inquire about *unique outcomes* and an alternative storyline about their lives (White 2007). A unique outcome (White and Epston 1990) refers to an event, action, or experience that contradicts the dominant or problem‐saturated story that a client tells about their life situation. It highlights moments where the person did not conform to the problem's narrative and upon which a therapist can invite the client to begin building a more empowering storyline about their life. The therapist assumes the unique outcomes are there, but they need to be perceived to be employed.

Similarly, in the domain of solution‐focused therapy, clients afford the therapist opportunities to inquire about *exceptions* to the problem (Berg 1994; De Shazer 1988, 1991). In a recent extension of solution‐focused work, Taylor and Simon (2014, 2024) claim that almost every client utterance affords the therapist opportunities for a solution‐building response. The solution‐focused therapist assumes that affordances for solutions already exist and simply need to be perceived to be utilized. In the domain of response‐based practice (Wade 2007a, 2007b), clients who claim to be offended by injustice or violence are seen as affording the therapist with opportunities to dignify the client by drawing out and acknowledging her agency in *resisting* that abuse. The therapist assumes that the client inevitably resisted in some way (at the time of the abuse) and that bringing forth a conscious awareness of that resistance gives the client a coherent basis for honoring and appreciating themselves for resisting (even if they were not successful in stopping the abuse).

And in the domain of narrative‐informed couple therapy (Madigan 2019), the specific history of the couple's early relationship when they first came together affords the therapist an opportunity to bring forth *common values and beliefs* that enabled the couple to create their unique relationship in the first place. Engaging in a selective historical inquiry at the outset of couple therapy provides a foundation for rekindling mutual respect and caring within which potential therapeutic changes might be explored more safely. In all these examples, pre‐existing positive affordances are already available and provide opportunities for therapists to take specific therapeutic initiatives. However, they need to be perceived and privileged by the therapist before they can be utilized in therapy. Hence, Gibson's general assumption about the ecological “already thereness” of affordances is relevant.

To support an understanding of “relational affordances” available in therapy, we offer the concept of contextualized couplings or complementarities. Gibson said he coined the term affordance to represent the *interaction* between the animal and the environment:

The *affordances* of the environment are what it *offers* the animal, what it *provides* or *furnishes*, *either for good or ill*. The verb to *afford* is found in the dictionary, but the noun affordance is not. I have made it up. I mean by it something that refers to both the environment and the animal in a way that no existing term does. It implies the complementarity of the animal and the environment.

(Gibson 1979, p. 127)

For our purposes in family therapy, at least two distinct complementarities are in play. One is *clients in their natural environment*, which includes other people, other animate beings, and inanimate objects. The other is *therapist in the environment of therapy* which includes clients, clinical team members, history, and preconceived beliefs brought by all involved people as well as the physical setting. In other words, there are relational affordances to attend to in both the “family system” and in the “therapy system.”

Regarding the coupling of clients in their natural environment, we maintain the assumption that their environment presents both life‐supporting and life‐threatening affordances; and thriving depends on perceiving and accepting life‐supporting possibilities as well as declining life‐threatening ones. The therapist in the therapy environment coupling will also encounter a range of affordances, some more potentially supportive of co‐constructing useful change than others, depending partly on the interviewer's model of practice. “Thriving” in the therapy system depends on utilizing those affordances that favor wellness in the family system. The mindset of an affordance‐oriented interviewer may be guided by the operative question: what are clients bringing to the process that may be collaboratively highlighted to enhance relational wellness? A more deficit‐oriented interviewer may be guided by a different question: what is missing or broken that I must supply or repair? It seems there would be significant intersection points between these two couplings. Therapy could be construed as an activity designed to help clients more successfully access the positive affordances in their environment and more successfully decline the negative affordances.

### 2.3 Affordances Becoming Opportunities

Affordances and opportunities are closely related concepts, but they can be usefully differentiated to provide somewhat different meanings. Both are distinctions that can be brought forth by an observer (who, in our case, would be the therapist). Affordances refer to the *perceived* or actual capabilities of an environment and what interactions are possible. Opportunities represent a broader cognitive phenomenon: they are more conceptual and include the perceiver's desires, intentions, and/or hopes for a preferred outcome. To be sure, opportunities also depend on perception, but they are proactively oriented toward *conceiving* of circumstances or situations that lead to positive outcomes (such as cooperation, collaboration, problem‐solving, solution‐building, synergy, etc.).

A significant advantage in differentiating affordances from opportunities is that one can then foreground the realization that affordances already exist, even when they are not being perceived, whereas opportunities arise only when an observer conceives of some benefits from utilizing an affordance that has been perceived. What is unique in recognizing an opportunity is the positive intention of the observer in distinguishing and using it. Affordances may be either positive or negative, depending on how they are evaluated. However, opportunities (in the context of doing therapy) are positive by definition, whether they are utilized or not.

The assumption of “already‐thereness” of affordances is extremely important. If one believes that a certain affordance already exists, it is more likely that one will look for it, and if one looks, it is far more likely that one will find it. Indeed, simply entertaining the notion of affordances could be expected to bias the observer to look more closely into the environment or niche to perceive its potential affordances. In this paper, we are inviting all therapists to embrace the concept of affordances and are encouraging them to lean more heavily into stronger habits of looking for the kinds of affordances that clients bring with them to therapy. The reason is that once an affordance is recognized and understood, the possibility of the observer being able to construct a realistic opportunity out of it is greatly enhanced. We have already alluded to the ways in which narrative therapists, solution‐focused therapists, response‐based therapists, and narrative‐informed couple therapists already do this implicitly. One purpose in writing this paper is to become more inclined to identify therapeutic affordances that are already there. A second purpose is to clarify the sequence of therapist activity from perceiving affordances to formulating opportunities to taking the initiative to respond therapeutically. Much of this sequence has already been worked out in solution‐focused therapy (Simon and Taylor 2024) and will be reviewed later. But first, let us focus on affordances for reciprocal interaction in relationships.

### 2.4 Affordances, Opportunities, and Initiatives Relevant to *Relational* Wellness

Over the past 35 years, Tomm and his colleagues at the Calgary Family Therapy Centre developed the IPscope, a systemic perceptual–conceptual tool, to distinguish different kinds of relational interaction patterns (Tomm 2014). Therapists can use this instrument to generate selective awareness about what might be taking place in the relational space by simplifying the enormous complexity of interpersonal interaction as family members engage in ongoing relationships with one another. For example, a behavior of criticizing typically invites a behavior of defending, and the defending then typically invites further criticizing, reflecting a reciprocal complementarity in their natural environment. These behaviors tend to become *coupled* as a recurrent interactional component of the relationship. Within this common pattern of “criticism coupled with defensiveness,” a critical comment *affords* the possibility for a defense and the defense in turn *affords* the possibility of further criticism. Even though the pattern is not wanted, and the negative affordances need not be taken up by the participants, they often are, simply because the reciprocity in the coupling becomes so habitual. The participants are very familiar with these mutual affordances and readily re‐enact the relevant behaviors to reproduce the problematic pattern again and again without realizing they are actively maintaining the problem. Needless to say, whenever this kind of Pathologizing Interpersonal Pattern (PIP) comes to dominate a relationship, both participants end up living in unwanted misery.

The good news is that these same participants always bring positive affordances to their relationships as well. Living in patterns of mutual caring and loving respect is a foundational requirement for the survival of human beings (Maturana and Varela 1987; Maturana, Verden‐Zoller, and Bunnell 2009). Affordances to enact positive interaction patterns are always part of our social niche. The manner in which these various relational patterns unfold in families and other human systems varies enormously. The IPscopic framework posits that, over time, every family as a relationship system develops a repertoire of different kinds of patterns of interpersonal interaction (Tomm 2014), which become part of their local culture. This repertoire includes affordances not just for PIPs but also for Healing Interpersonal Patterns (HIPs) and Wellness Interpersonal Patterns (WIPs). The potential to re‐enact any of these pre‐existing relational patterns is already there. For instance, a behavior of acknowledging the other readily *affords* a response of appreciating such an acknowledgment, and the appreciation, in turn, *affords* further acknowledgment. Prior familiarity with this wellness pattern of “acknowledging coupled with appreciating” affords the possibility of it being brought forth within the therapy system to be enacted in the family system. A common healing pattern includes “expressing regret coupled with accepting the regret.” An even more powerful relational healing pattern entails “apologizing coupled with forgiving,” which most families establish to some degree as part of their repertoire. It, too, is usually available as an affordance. In other words, positive affordances to engage in healing and wellness patterns exist alongside negative affordances. Being mindful of these pre‐existing positive possibilities in the culture of the family system increases the probability that therapists will perceive them and then utilize them in therapy. For example, if one family member is encouraged by a therapist to respond to a critical comment by another family member with an acknowledgment of the caring concern behind that comment, an entirely different pre‐existing relational pattern might be brought forth.

While the good news is that clients always provide affordances for therapists to be therapeutic, the bad news is that therapists often do not see or hear them and, hence, miss opportunities to utilize them. Our job as systemic therapists is to become skilled enough to perceive the possibilities of positive affordances, transform them conceptually into opportunities for more relational wellness, and then take initiatives to enable family members to take up the opportunities and respond to one another in preferred patterns of interaction.

## 3 Opportunities

### 3.1 Opportunities in the Context of Solution‐Focused Therapy

Solution‐Focused Brief Therapy was developed by Insoo Kim Berg and Steve de Shazer along with their colleagues at the Brief Family Therapy Centre in Milwaukee, Wisconsin (De Shazer 1985, 1988). A key premise of solution‐focused practice that crystallized over time is that the problems people bring to therapy will exhibit exceptions—that is, times when the problem could happen but does not—and that these exceptions contain clues to solutions. In other words, clients will almost always afford the therapist opportunities to perceive exceptions to their problems, and these exceptions will provide both the client and therapist with opportunities for solution‐building.

Around the year 2000, several members of the international community of solution‐focused practitioners became aware of a team led by Janet Bavelas at the University of Victoria (Beavin‐Bavelas 2022) who, for decades, had studied face‐to‐face dialog using the tools of conversational microanalysis. Among them, Taylor and Simon (2014) began a study of *keywords* in therapeutic conversation. The central conclusion of this work (expanded recently in Simon and Taylor 2024) is that client statements in therapy contain multiple opportunities for solution‐building responses by the interviewer and that for each perceived opportunity, there will also be multiple possibilities for a therapeutic response. An expanding awareness of these opportunities and possibilities sets the stage for the selection of a preferred opportunity–response pairing as a therapeutic response to the original client statement. Some opportunities are perceived by the interviewer to be more promising than others. Some affordances presented by clients are considered by the therapist to offer a more direct path to wellness at that point in the interview. The utility of this awareness lies in orienting the interviewer's attention to specific client language and making considered choices of responses step by step through the interview. The starting point for Taylor and Simon was to recognize the opportunities in the client's utterances. The presence of affordances in this formulation was implicit but was not recognized as such.

### 3.2 An Extension of Solution‐Focused Work Into the Concept of Affordances

In this article, we are extending the model of the opportunity–response sequence by adding the element of clients first *affording* the therapist certain possibilities in the client–therapist system. As noted earlier, clients bring pre‐existing affordances for possible therapeutic change with them to the therapy. These affordances are the foundation upon which the opportunities are built. One generic pre‐existing affordance would be the client's (implicit or explicit) hope that something in their situation could improve, or at least things could be different. Another is the hope that the therapist could bring something to the situation that might be helpful. Exactly how these hopes become manifest in a specific therapy varies a great deal, but they are always there. Perhaps the simplest and most straightforward manner in which these affordances may be revealed is in the specific *words* clients use while sharing their experiences during the therapy conversation. Certain words, phrases, sentences, and associated nuances afford the therapist unique opportunities for a therapeutic response.

Opportunities do not spring up out of nowhere. They are grounded in the affordances that the clients bring to therapy *and* in the imagination of clients and/or therapists about what could be different. Once an affordance has been perceived and an opportunity has been conceived, the therapist may proceed to construct a response. One schema for response construction is known as the *preserve–omit–add* procedure (Korman, Bavelas, and De Jong 2013). In constructing a verbal response to the client, therapists “preserve” a keyword or phrase from a client statement, “omit” the rest of the statement, and then “add” words with therapeutic intent. The following excerpt of therapeutic conversation (Simon and Taylor 2024, 77) illustrates such a response construction:

Therapist: Let's say you woke up in the morning … without this negative outlook, how would you start catching on this is different?

Client: Um. rather than waking up and maybe hurriedly going through like my morning routine, maybe I'd just take my time and relax a little bit more. There's a pressure in ‐ like all the time ‐ so, maybe I'd just be a little bit more calm.

Therapist: Ya. Okay. And, since you started out the day that way—more relaxed, going a little bit slower, more calm ‐ how would the day go differently?

In this excerpt, the therapist draws upon the client's pre‐existing affordance of hopes for something different and opens space for the client to reflect (using an “imagine if” or “suppose” question). The client reveals the specific hopes of more relaxation and calmness. The therapist seizes upon the opportunity to formulate a therapeutic response by preserving the client's hopeful words, deleting the other words, and adding “How would the day go differently?” to elicit details of difference and invite “thickening” of possibilities in the preferred direction.

Rehearsing this sequence of perceiving affordances, conceiving opportunities, and generating therapeutic responses could significantly strengthen any therapist's skills. This process could be described as *opportunizing* (Christiansen 2005) and will be demonstrated below in some clinical examples under the section on therapeutic initiatives. Suffice it to say here that opportunizing entails taking up an affordance and conceiving of it in a way to deliberately guide clients' interactions within their niche toward preferred outcomes.

To take the assumption of the “already‐thereness” of affordances a bit further, we would like to highlight the availability of *immanent affordances* that a therapist can draw upon. As with the hopes that clients bring to therapy, immanent affordances are presupposed by the therapist and are presumed to be available even when they are not yet manifest. They are more complex than the *surface affordances* that arise from what clients actually say or do in the session. Immanent affordances are embedded in a rich history of memories, internalized cultural values and beliefs, and prior relational experiences. These understandings can proactively be brought forth as affordances from this background of prior knowledge and foregrounded by the therapist in the here and now of the current therapeutic conversation. For instance, the relational healing affordance of forgiveness may not be perceived by family members during an active conflict, but it could potentially be drawn out of latency and brought into focus by the therapist because they are already familiar with the relational process of forgiving. In doing so, however, extra care needs to be taken to remain attuned (to listen to the readiness of the client to perceive the affordance) and ensure that there is sufficient commonality in the meanings, understandings, and utterances being used by both clients and therapists to make the immanent affordances become evident in a coherent manner.

## 4 Therapeutic Initiatives for *Relational* Wellness

As affordances are offered by clients, with or without conscious awareness, and therapists conceive of potential opportunities for more wellness, they collaborate in a process of co‐constructing preferred possibilities. In this collaboration, a systemic therapist is neither passive nor neutral. She is typically very active in asking clarifying questions to disclose relevant affordances that may be available, transforming them into opportunities, and then taking the initiative to help clients benefit from those opportunities. And while she may be neutral about the specific goals a client may be striving toward, she is always ethically biased toward movement in *a constructive direction*, namely, toward greater wellness. This can become rather challenging at times, especially in conjoint family work. The goals of various family members often differ, and a constructive direction for one family member may not be constructive for another family member. It is for this reason that we prefer to focus on prioritizing *relational* wellness as much as possible, given that, in our experience, more individual wellness for the participants will follow. In other words, whenever possible, we privilege an opportunity–response pairing where all parties are more liable to benefit. And this is where the IPscope becomes a valuable resource in therapist decision‐making. The intervention that is most likely to activate or bring forth a healing pattern (HIP) or a wellness pattern (WIP) is given priority. Every family situation is unique with respect to which specific HIPs or WIPs the therapist might perceive and choose to invoke. It is possible, however, for IPscope‐informed therapists to develop better skills in perceiving and distinguishing the more promising HIPs and WIPs that clients afford therapists, as well as opportunities that could be brought forth in any particular family situation at any particular time. It is beyond the scope of this paper to attempt to describe the myriad of interventive options that could be used to do so in systemic therapy. We will limit ourselves here to emphasizing the sequence of affordances followed by opportunities and give a few examples of therapist initiatives in the following vignettes of therapeutic conversation arising from the authors' clinical practices.

## 5 Clinical Examples

### 5.1 Vignette #1—Movie‐Making Sisters

In a recent parenting interview with a mother and father of two girls, aged 9 and 12 (not present in the interview), the parents identified “constant bickering” between their daughters as the primary concern. They suggested that the older girl, Judy,^1^ was the instigator of “picking at each other” and the source of “constant complaining” that frequently “totally ruined days for the whole family at home and on outings.” A tone of discouragement accompanied the parents' replaying of examples of problematic interactions. Almost as a component of the complaints, the mother said: “the only time the girls behave is when we are holidaying in Hawaii, when we have company, and when they are both in trouble for misbehavior.”

Therapist: Can you pick one of these times when you had company, or the girls were in trouble.

Mother: Well … yesterday … and they ended up going downstairs to make a movie.

Therapist: How long were they down there making a movie?

Mother: A half hour … or maybe 45 minutes.

Therapist: So how do they make movies?

Mother: I don't know … it's an app … or something like that.

Therapist: Who suggested the movie project?

Mother: Judy [eldest daughter].

Therapist: If I had a scale where 10 was getting along well and 0 is the opposite, where would you rate the girls interacting yesterday, making the movie?

Father: Oh … nine.

Mother: Or even nine and a half.

Both the interviewer and an observing colleague noticed a clear shift in the parents' demeanor after this exchange. They brightened in facial expressions and tone of voice. They relaxed and began to interact cooperatively to produce useful ideas. One might characterize this movement as a generative shift from the language of complaint and hopelessness to one of hopefulness and possibility, judging by the subsequent comments we heard:

– “Maybe we've been focusing too much on the negative.”
– “We could try agreeing on a strategy for dealing with the bickering and then stick with it for a month or two instead of just a couple of days.”
– “We could ask them to show us the movie they made.”

### 5.2 Commentary on Vignette #1

The revelations by the parents of times when the problem does not happen are classic examples of *exceptions to the problem*, which are a core element in solution‐focused practice. They were offered spontaneously, without request or specific invitation by the interviewer, as is often the case. In disclosing these exceptions, the parents afforded the therapist an opportunity to selectively highlight a constructive relational process between the sisters. In terms of opportunities analysis, these exceptions to the problem provide a basis for an opportunity for solution‐building. The exceptions afforded the interviewer the option to selectively focus on the parents' words, to engage in detailed questioning to recall the sense of this being a real occurrence, and to employ a scaling question (Berg 1994; De Shazer 1994) as a tool to magnify the experience of success. The interviewer's solution‐building responses afforded the clients an opportunity to pivot from complaint to possible solutions, an embedded offering that the clients appear to have taken up, judging by their subsequent more hopeful and generative comments.

It is interesting to reflect on the relational patterns of interaction in this excerpt. Early in the conversation, the mother and father exhibited a coupling of complaining (about their daughters' bickering) and compounding the complaints (by adding additional examples of misbehavior to what the other parent mentioned). The “complaining coupled with compounding” pattern afforded the therapist an opportunity to enter more deeply into the parenting disappointments and difficulties. Fortunately, this negative affordance was not taken up. Instead, the therapist took up the positive affordance of engaging with the important exception of movie making. As a result, a more healing pattern emerged as imagining possibilities for alternative understandings and guidance of their daughters' behavior coupled with affirming possibilities suggested by the other parent. The HIP of “imagining coupled with affirming” potentially positioned the parents to engage with their children more positively when they returned home.

### 5.3 Vignette #2—Enabling a Shift From Pressuring to Forgiving

A repentant father sought therapy as part of an effort to reconcile with his 11‐year‐old son, who refused to visit the father's home after the parents separated. There was a history of some prior physical abuse during drinking episodes when the boy did not comply with parental guidance. The father had stopped drinking since the separation and had apologized to the son for his past abusive behavior. However, the son still refused to go to the father's place despite the mother actively encouraging him to do so.

Father: I want to spend more time with him. I don't know what to do. I've stopped drinking, I've apologized, I've promised to give him full access to his phone, but he still won't come.

Mother: His Dad really has made a lot of changes for the better, and I think they need more time together.

Therapist: It sounds like both of you are strongly in favor of a good father‐son connection. (Turning to the son) Are you feeling some pressure to spend more time with your Dad?

Son: Yeah.

Therapist: Does the pressure make it easier or harder to accept his request?

Son: Harder.

Father: I think he still resents me for hitting him when I was drinking. Why can't he just forgive me for the mistakes I made so long ago? I've apologized so many times!

Therapist: Say he wasn't able to let go of his memories of the past, do you think it might be possible for you to forgive him for not yet forgiving you?

Father: (after a pause, and with a softer tone) Oh, I hadn't thought of that. … Maybe I could …

Son: (speaking up spontaneously to the father) I'm sorry, I really do want to go to your place, but I'm still a bit too scared …

### 5.4 Commentary on Vignette #2

In this example, the therapist perceived the *intensity* of the father's desire to reconcile as blocking what he was hoping for. The intensity came across as pressuring the boy to comply. In doing so, it constituted a negative affordance. Pressuring typically affords resisting, and resisting typically affords more pressuring. In other words, the pathologizing interpersonal pattern (PIP) of “pressuring coupled with resisting” appeared to be active in the relationship despite the parents' genuine efforts to escape it. The therapist recognized the opportunity to invite the family to reflect on this pattern and become aware of the negative affordances. He did so by asking the boy some questions to open space for him to disclose his experience of pressure and his reaction of resisting. The hope, of course, is that with increased awareness of the consequences of choosing to take up negative affordances, family members would try to orient themselves to avoid utilizing them.

The therapist also perceived the family's familiarity with the interaction pattern of “apologizing coupled with forgiving” which is ordinarily very positive and affords healing in wounded relationships. In this case, the negative affordance of intensity inadvertently used a potentially positive affordance in the healing pattern to serve as a tool in the pressuring process (he *should* forgive me). Neither parent appeared to recognize how the pressure from the father for the son to forgive was actually maintaining the PIP. The therapist recognized the opportunity to harness the positivity in the forgiveness affordance by inviting the father to shift his location within the healing pattern (of apologizing coupled with forgiving) so its desired effects could be realized. He was able to achieve this by asking the father a reflexive question (Tomm 1987) about whether he could forgive the son for not yet being able to forgive him. When the father acknowledged this possibility and began considering the option of moving from apologizing to forgiving, the boy assumed the complementary position in the same relational healing pattern and spontaneously began apologizing.

### 5.5 Vignette #3—Liberation From Oppressive Silencing

A 22‐year‐old woman was referred with her family for therapy when a nurse noticed significant tension in the family during a follow‐up appointment at an out‐patient clinic after she had been hospitalized for “psychosis.” The young woman lived with her parents and a younger brother. A few years earlier, she had had a major falling out with some friends and had dropped out of university. She took no initiative to obtain any gainful employment and withdrew from all social interaction. The parents insisted the daughter accompany them to the first interview.

Therapist: What are your hopes in coming for family therapy?

Mother: We're feeling desperate.

Father: Whatever helps. …

Therapist: (to daughter) How do you feel about being referred for family therapy?

Daughter: (opens her mouth as if to speak but suddenly stops, closes her eyes, drops her head, and then falls silent.)

The mother proceeds to volunteer detailed information about the daughter's past mental difficulties, psychiatric admissions to the hospital, several investigations, and multiple therapies. The mother becomes very tearful in the process. She goes on to describe how the daughter stays in her room almost all the time and never does any chores. The father expresses frustration in not being able to get the daughter to do anything. He describes how he has to force her out of her room to take their meals together. While the parents are talking, the daughter appears to tune out and remains totally unresponsive.

Therapist: (trying to shift the focus away from the daughter onto the parents) What happens to you as parents when you see her shutting down like this?

Mother: We are worried sick that she might have some kind of dementia.

Therapist: What effect do you imagine our talk about your worries might be having on her?

Mother: I don't know what is going on for her. She seldom says anything.

Therapist: Who would be the first to notice small steps she might take to express herself?

Father: I notice that she sometimes gets annoyed and swears under her breath when I try to force her out of her room.

Therapist: How do you respond to her swearing?

Father: We're a Christian family and don't accept any vulgar language.

Therapist: Can you imagine that if she were allowed to express her frustration openly and freely, instead of keeping it inside, you might get a little window on what's going on inside her?

Father: Maybe. …

Therapist: I wonder if she may have internalized some Christian values to an extreme—maybe to the extent that she is now being oppressed by a prohibition against swearing, which is forcing her into silence. Would you be willing to experiment with an opposite approach, at least temporarily, and actively encourage her to yell and scream whenever she seems frustrated, to see what happens?

Mother: We're willing to try anything.

After the session, the parents took up the therapist's suggestion and actively supported the daughter in expressing her anger. She gradually opened up both at home and in therapy. Indeed, in subsequent sessions, she explicitly challenged her parents' “excessive worries” and their “intrusiveness.” After a few months, she was well enough to make new friends and register for a course at the university.

### 5.6 Commentary on Vignette #3

The family afforded the therapist an opportunity to observe a pathologizing relational process of “IP (identified patient) gazing” in the first session whereby “worrisome scrutinizing coupled with withdrawing and distancing” appeared to be aggravating an already difficult situation. The therapist may have inadvertently added to this pathologizing pattern by asking the daughter about her feelings about therapy at the outset, that is, to participate in “self‐scrutinizing.” Based on an assumption that there must be some positive affordances available for connecting with others, the therapist asked for small initiatives she may have taken to communicate (while shifting the focus off her and onto who might notice). This yielded the opportunity to imagine a healing pattern of “encouraging the open expression of anger coupled with taking risks in expressing herself more freely,” which was introduced as a possible experiment. The family took up this opportunity and ultimately was able to enter into a more stable relational wellness pattern of “parents giving more space for free expression of emotion coupled with daughter risking more open and honest self‐expression.”

### 5.7 Vignette #4—A Little Kindness and Consideration

This example is a little different in that it includes a supervisory process with an illustration of a perceptual prompt. At the onset of the pandemic, the Calgary Family Therapy Centre (where we work) ceased to provide face‐to‐face services. Instead, we initiated online therapy to continue serving our clients. The Centre practices close supervision of interns, generally favoring live coaching of sessions. In exploring possibilities for a virtual analog for that aspect of our practice, we began to use the chat function on our virtual meeting platform to communicate with therapists privately during the actual session. For those therapists who were able to manage multitasking, we found that this technical option afforded immediate and substantive feedback to interviewers throughout the session.

As a sidebar, it is interesting to note some of the content of the supervisor's live messages to student interviewers:

- Supervisors note and affirm selective therapist actions with a “thumbs up” or “check mark,” an emoji, a short phrase like “good question” or “nice waiting.”
- They recommend useful understandings, such as “sounds like a normal part of living” or “I'm hearing beginnings of agreement here.” We have found that this kind of comment helps student interviewers perceive more client affordances.
- Supervisors might also suggest interventions such as questions like “how did they do that?” or “who benefitted from that effort?” This helps interviewers activate opportunities with options for responding therapeutically.

A brief account of a recent session exemplifies this procedure in action. An intern was meeting with two parents of a family designated as “high discrepancy;” a description used at the Centre to denote persistent relational couplings that exhibit strong differences that often escalate into robust conflict. The father had left the family home recently and was initiating divorce proceedings. The mother expressed “shock and hurt” at this “sudden development.” The parents presented with shared concerns about the impact on their children. The father “hoped to see his daughter” who was refusing to see him, declaring that she “needed time to heal.” As the interview progressed, one of the parents, by tone or choice of words, would repeatedly irritate the other, who would respond in kind, with the interaction quickly deteriorating into a PIP of “blaming coupled with counter blaming.” This pattern had the effect of distracting from and overshadowing the central hope of the parents and, indeed, the mission of the Centre, namely, to nurture the health of the children.

The intern interviewer was doing well with these challenging circumstances, allowing for some authentic expression of hurt and resentment, and then intervening to create a “pause” to “let feelings calm down” and “get back to the future health of the children.” At one point, the mother said in exasperation:

Mother: All I'm asking for is a little kindness and consideration.

Father: Well … there are things to attend to … with house … and bills …

[As the conversation continued argumentatively, in the direction of these instrumental tasks and responsibilities, the supervisor sent a private chat message to the intern “she said kindness and consideration.” The interviewer noted the supervisor's comment and soon put the perception to use.]

Therapist: Mary, you mentioned kindness and consideration …

Mother: Ya … some of the emails telling me what to do … blaming me for turning the children against him …

Father: [After a thoughtful pause] I know … emails … sometimes people take things the wrong way … and maybe I could be more careful … how I say things … I just really want to see my daughter … when I say something hurtful, could you tell me? …

Mother: I want the kids to be able to see their dad … as long as they feel safe …

Father: Whatever that takes … even Jane (therapist) or someone else being present …

### 5.8 Commentary on Vignette #4

One way of understanding this exchange and the generative shift that occurred during the session could be that with the live coaching of the supervisor, the student interviewer was able to perceive the positive surface affordance in the mother's words, “kindness and consideration.” She was then able to couple it with the immanent parental affordance of common striving to care for and connect with the children. The mother, in this case, brought the affordance of preferring kindness into the conversation with her phrase “kindness and consideration.” Despite the fact that her comment arose as a component of complaint about negativity in interaction with her estranged husband, it could be perceived as positive and was successfully put to work as an opportunity for shifting their relationship toward wellness, even though the circumstances were very difficult.

## 6 Concluding Comments

We would like to conclude by emphasizing the responsibility that we have as therapists to work toward the well‐being of our clients. Clients come to us with their troubles and vulnerabilities, and they trust us to respond in ways that could be helpful. To be ethical practitioners, our intended responses need to be aligned with their hopes and toward greater wellness. In addition, we need to endeavor to empower them to be able to continue toward more wellness outside of therapy and not become dependent upon us. Improvements are more liable to be maintained when we intervene selectively to privilege reciprocal couplings of wellness in the clients' natural niche of relationships. Consequently, it is incumbent upon us to be very intentional in what we do and do not do in doing therapy.

Our belief is that if we, as therapists, become more rigorous in recognizing and enacting preferred sequences in the therapy process, the odds are greater that we will be more successful in enabling our clients' wellness. There are many ways that we can become more disciplined in our work as therapists. The kind of rigor that we are encouraging in this paper is to engage in recurrent sequences of perceiving client affordances for relational healing, constructing opportunities for preferred interaction patterns, and taking initiatives to enable a realization of those opportunities. As noted earlier, the already‐thereness of affordances gives us confidence in seeking to perceive them. Our intention to support greater relational wellness gives us directionality to conceptually convert selective affordances into constructive opportunities. Whenever possible, we encourage the selection of opportunities that fit into reciprocal relational patterns, which build more endurance and stability in the relationships of the family system. And finally, our passion for helping our clients, mobilizes us to take the initiative to open space for them to take up the positive affordances that are available to them.

This is not to say that we are against intuitive spontaneity in the doing of therapy. We believe intuition can be expanded when deliberate efforts are made to reflect on our practices and enhance our awareness of possibilities. For instance, if, while reading this paper, you become inclined to orient yourself to perceive the positive affordances that your clients bring to your practice, you might more readily notice them in your future sessions. And if you learn to receive these affordances as “gifts” from your clients to support your work, you will become even more inclined to reciprocate with interventions to utilize these therapeutic opportunities.

## Endnotes

1 The name has been changed to protect the client's privacy.
